# Patients’ and Doctors’ Beliefs about Treatment and Long-Term Adherence in Rheumatic Diseases

**DOI:** 10.31138/mjr.31.1.152

**Published:** 2020-06-11

**Authors:** John Yfantopoulos, Marianna Protopapa, Konstantinos Mantalias, Athanasios Chantzaras, Katerina Koutsogianni, Platonas Yfantopoulos, Dimitrios Vassilopoulos

**Affiliations:** 1 Professor of Health Economics, MBA-Health, National and Kapodistrian University of Athens, Greece; 2 MBA-Health, National and Kapodistrian University of Athens, Greece; 3 President of the PanHellenic Federation of Patients, Parents, Caregivers and Friends of Children with Rheumatic Diseases, RHEUMAZEIN; 4 University of York, United Kingdom; 5 Professor of Medical Rheumatology, School of Medicine, National and Kapodistrian University of Athens, Greece

**Keywords:** Medicine adherence, rheumatoid arthritis, communication between the patient and the doctor

## Abstract

**Objective:**

The aim of this study was to explore the beliefs of rheumatologists and patients about treatment-related factors, long-term adherence, and their communication with regard to rheumatic diseases.

**Methods:**

In a multicentre, observational study conducted in Greece, a structured questionnaire was administered to 75 rheumatologists and 398 rheumatic patients from different regions. Five domains were investigated: i) effectiveness of treatment, ii) choice of treatment, iii) change of ineffective treatment, iv) long-term adherence, and v) the quality of communication between doctors and patients. Descriptive data, confidence intervals, t-tests and factor analysis were employed.

**Results:**

Examining the patients’ and rheumatologists’ beliefs and attitudes about treatment profiles and long-term adherence, a statistically significant convergence in their views on effectiveness and safety as the predominant factors concerning choice of treatment and long-term adherence was found. Although patients reported high trust to their doctors, a divergence of views is recorded regarding communication of the two parts. Statistically significant differences in the views between patients and rheumatologists were found with regards to access (p<0.001), time per visit (p<0.001), mutual understanding (p<0.001), and overall communication (p<0.001).

**Conclusions:**

Our study shows a great rate of agreement between patients and rheumatologists regarding the factors determining the efficacy, choice, switching and adherence to treatment while there was significant divergence in the views regarding the quality of communication between the two parts. Co-ordinated efforts are needed in order to improve the communication level between rheumatic patients and rheumatologists.

## INTRODUCTION

Chronic rheumatic conditions comprise over 150 diseases and syndromes, which are usually progressive and associated with pain.^1^ In short, this group of conditions includes rheumatoid arthritis, osteoarthritis, spinal disorders, and severe limb trauma. For the purposes of this study, we mainly focus on patients with rheumatoid arthritis. Rheumatoid arthritis (RA) is a chronic, incapacitating,^1^ inflammatory arthritis that results, without appropriate therapy, in joint destruction and reduced quality of life and working ability.^2–8^ It has often been argued that RA treatment choice should be based on what is often referred to as “shared decision-making”^9,10^ between the patient and the rheumatologist. Szasz and Hollender^11^ suggested that the most appropriate type of doctor-patient relationship is the “mutual participation” model. This implies that both parties share responsibility for planning and implementing treatment. Hendrikx et al.^12^ proposed an “asking and listening to the patient” model, emphasizing patient’s satisfaction with RA therapy leading to pain relief and less anxiety.

However, there is a discrepancy in the doctors-patients views in physical functioning and health in general.^13,14^ Doctors are more knowledgeable about the factors contributing to health, the clinical effectiveness, and the potential risks associated with a treatment profile compared to their patients.

The literature has indicated that, the most commonly cited barriers in the doctors-patients relationship are i) health literacy,^15–17^ ii) adverse effects,^18–22^ iii) poor communication on doctors’ part,^22–25^ iv) lack of trust of the doctor,^23,26,27^ v) cost or lack of insurance coverage,^19,20,22,24,26,28–31^ and vi) changes to the medication that affect patients’ routine.^19,21,24,28^

In its 2003 report on medication adherence, the World Health Organisation (WHO) quoted the statement by Haynes et al.^32^ saying that “increasing the effectiveness of adherence interventions may have a far greater impact on the health of the population than any improvement in specific medical treatments”.^33^ According to the same report, adherence rates in developed countries rates averaged around only about 50%. It should be noted that adherence is strongly associated with the effectiveness of all therapies.

Medication adherence is related to three major groups of factors: i) patient characteristics and health status, ii) disease features, and iii) treatment effectiveness. A great part of the literature focuses on the patients’ barriers to adherence. It has often been argued that patients’ non-adherence is related to several factors, such as: i) lack of information concerning the clinical effectiveness of alternative treatment profiles,^34^ ii) fear of side effects,35 and iii) limited communication between patients and doctors.^35^

Doctors have a substantial role to play in improving the communication with their patients by sharing information on biological, psychological, behavioural and social aspect of their patients’ health^36,37^ and providing both verbal and non-verbal guidance^30,38^ in order to achieve the desired results.^38,39^

The purpose of this paper is to investigate patients’ and rheumatologists’ beliefs about treatment related factors, long-term adherence, and the communication between them concerning rheumatic diseases.

## METHODS

A structured questionnaire was designed based on international literature to investigate beliefs and attitudes of rheumatologists and patients concerning the treatment-related factors, patients’ adherence, and the communication between the two parties. This questionnaire was administered to 75 rheumatologists and 398 rheumatic patients from different regions of Greece. A convenient quota sampling procedure was adopted, ensuring the representativeness in both samples with respect to sex, age, and other socio-demographic characteristics. In the patient group, adults were enrolled if they were above 18 years old, reporting suffering from an inflammatory rheumatic disease (rheumatoid arthritis-RA, ankylosing spondylitis-AS, psoriatic arthritis-PsA). The PanHellenic Federation of patients, parents, caregivers, and friends of children with rheumatic diseases associations (REUMAZEIN) organised the selection of patients’ data. The Web-Rating Health electronic tool was used to collect information on rheumatologists’ views in collaboration with the Greek (Hellenic) Society of Rheumatology. Given that disease management is a multifactorial procedure, five major dimensions have been selected to examine the attitudes and beliefs of rheumatologists and rheumatic patients about the treatment of the disease. The first dimension focuses on the effectiveness of the treatment in relation to pain relief, swelling, mobility, fatigue, lab tests, and manifestation of the disease. The second domain examines the choice of treatment with reference to the safety profile of drugs, access to and experience in clinical use of drugs. The third dimension investigates the changes of an ineffective treatment. The fourth dimension analyses the aspects of long-term adherence. Finally, the fifth domain concentrates on the doctor-patient communication. A series of five-level Likert items were used to examine subjects’ preferences, with the following possible responses: 1. Not at all, 2. A little, 3. Moderate, 4. Very, 5. Too much.

The Cronbach’s alpha test was applied to analyse the internal consistency in doctors and patients’ responses. The convergence or divergence in the views of these groups was examined and presented diagrammatically. T-tests were applied to examine the differences between the mean preferences of doctors and patients. Significance level was set at *α* = 0.05.

Factor analysis was performed for patients and rheumatologists to investigate the interrelationship between adherence and a set of constructs. The specified models are presented below:

Patient’s Long-Term Adherence = F {Safety, Effectiveness, Adjustment Maintenance , Cost, Agreement}

Doctor’s Long-Term Adherence = F {Safety, Effectiveness, Adjustment Maintenance , Cost, Agreement}

Factor analysis does not make *“a priori”* assumptions about the interrelationship of different constructs. Data analysis was conducted with the package SPSS v.26.

## RESULTS

The demographic and socio-economic characteristics of the rheumatologists and patients’ groups are presented in *Table 1*. The patient group consisted of 398 patients with inflammatory rheumatic diseases of which 72.1% were women. Around 51.6% of them suffered from RA, 27% from AS, and 21% from PsA. The mean age of patients was 53.74 years with a standard deviation of 14.72 years. The rheumatologists group includes 75 doctors of which 56% were females, with a mean age of 51 years (SD: 9 years).

**Table 1.** Socio-demographic characteristics of the participants.

		**Patients**		**Rheumatologists**		**Total**	
		**n**	**%**	**n**	**%**	**n**	**%**
**Sex**	Male	111	27,9%	33	44,0%	144	30,4%
	Female	287	72,1%	42	56,0%	329	69,6%
**Age (years)**	18–30	29	7,4%	0	0,0%	29	6,3%
	31–45	91	23,2%	22	30,6%	113	24,4%
	46–55	88	22,4%	27	37,5%	115	24,8%
	56–65	85	21,7%	15	20,8%	100	21,6%
	66–75	77	19,6%	8	11,1%	85	18,3%
	76–85	22	5,6%	0	0,0%	22	4,7%
mean (SD)		53.74 (14.72)		50.97 (9.01)			
**Type of rheumatic disease**	Rheumatoid arthritis	205	51,6%			205	51,6%
	Ankylosing spondylitis	107	27,0%			107	27,0%
	Psoriatic arthritis	85	21,4%			85	21,4%
**Occupation**	Employee	111	27,9%			111	27,9%
	Self Employed	60	15,1%			60	15,1%
	Student	10	2,5%			10	2,5%
	Household	57	14,3%			57	14,3%
	Unemployed	15	3,8%			15	3,8%
	Unemployed due to the disease	4	1,0%			4	1,0%
	Retired	141	35,4%			141	35,4%

The accepted Cronbach’s values for internal consistency is Cronbach’s alpha > 0.70.^40^ The Cronbach’s alpha test revealed a high level of internal consistency for both the rheumatic patients (Cronbach’s *α* = 0.918) and the rheumatologists (Cronbach’s *α* = 0.927).

### Effectiveness

**Figure 1.**
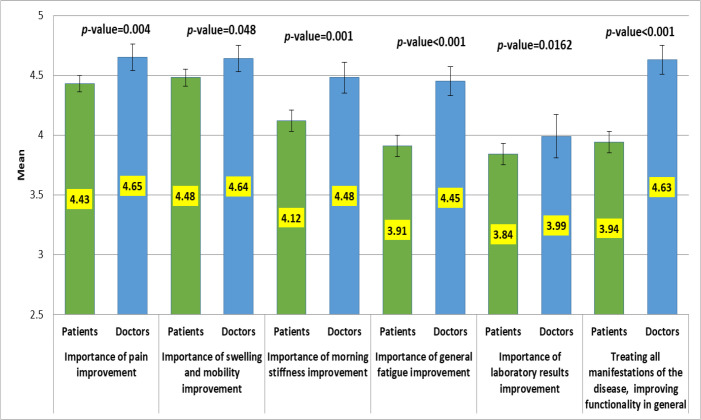
Importance of factors in determining the effectiveness of treatment. Note: p-values from t-tests of the mean difference between doctors and patients’ responses for each dimension.

The results about the mean perceived importance of each investigated factor in determining the effectiveness of treatment in rheumatic diseases, are displayed in *Figure 1*. Rheumatologists assigned greater values on average in comparison with patients for all items. All differences were statistically significant except for the laboratory results item: pain relief (*p* = 0.004), swelling and mobility improvement (*p* = 0.048), controlling stiffness (*p* = 0.001), fatigue (*p* < 0.001), laboratory results improvement (*p* = p= 0.0162), and treating all manifestations of the disease including functionality (*p* < 0.001). The largest differences in the mean responses between the rheumatologists and patient groups were observed in the items of: treating all the manifestations of the disease (mean difference = 0.69), general fatigue improvement (mean difference = 0.54) and morning stiffness (mean difference = 0.36). Interestingly, the lowest ratings for both groups were found in the laboratory results improvement factor.

It is well known that patients; views are influenced by subjective information such as pain relief,^41^ while doctors’ views are usually affected by biomedical factors such as swelling, mobility, stiffness, fatigue, and the overall functionality of the patient.^42^

### Choice of Treatment

**Figure 2.**
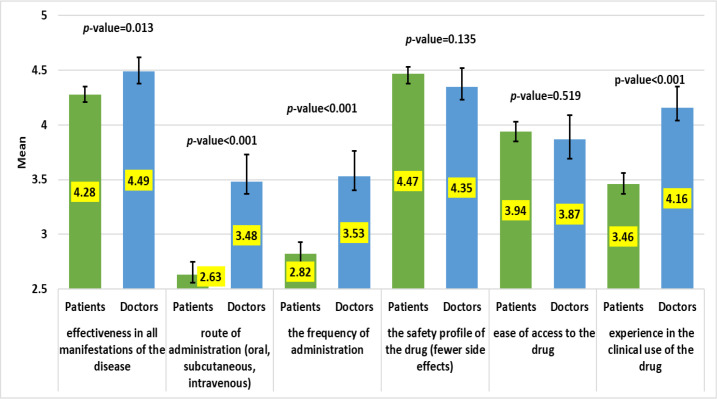
Importance of factors in determining the choice of treatment. Note: p-values from t-tests of the mean difference between doctors and patients’ responses for each dimension.

The results concerning the factors affecting the choice of treatment are displayed in *Figure 2*. Apart from the safety profile of, and the ease of access to, the drug, where responses were similar on average between the two groups, rheumatologists’ ascribed importance was higher on average for all other dimensions explored. The differences which were found to be statistically significant were: route of administration (*p* < 0.001), effectiveness in all of the manifestations of the drug (*p* = 0.013), frequency of administration of the treatment (*p* < 0.001), and experience in the clinical usage of drugs (*p* < 0.001). The route of administration (mean difference = 0.85) and the frequency of administration (mean difference = 0.71) were the items associated with the highest mean rating differences between the doctor and the patient groups. Patients seem to pay little attention to the route of their treatment’s administration as well as its frequency, while rheumatologists also rate these factors as the least important (*Figure 2*).

### Change of Treatment

**Figure 3.**
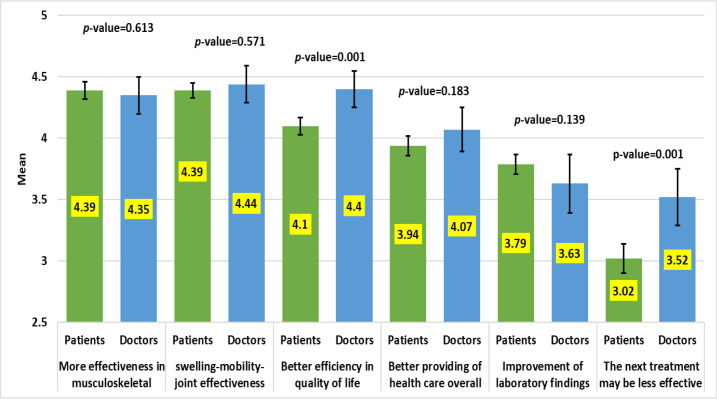
Importance of factors in switching treatment. *Note: p-values from t-tests of the mean difference between doctors and patients’ responses for each dimension.*

The change of an ineffective treatment is the third dimension that was explored (*Figure 3*). In most of the factors explored, a statistically significant difference was not found, except for the better effectiveness in quality of life (mean difference = 0.30, *p* = 0.001) and whether the next treatment would be even less effective (mean difference = 0.50, *p* = 0.001). Also, both rheumatologists and patients agree that the least important factor in this case is the possibility that the next drug would be even less effective.

### Long Term Adherence

**Figure 4.**
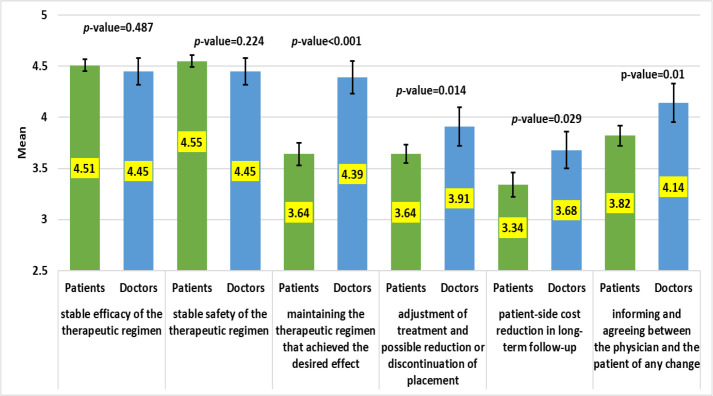
Importance of factors contributing to long-term adherence. Note: p-values from t-tests of the mean difference between doctors and patients’ responses for each dimension.

*Figure 4* displays the results concerning the perceived importance of factors contributing to long-term adherence. Both groups assigned high values to the lasting efficacy and safety of the therapeutic regimen as factors fostering long-term adherence (*Figure 4*). Rheumatologists recorded a statistically significant higher interest in maintaining the therapeutic regimen that achieved the desired effect, compared with patients (mean difference = 0.75, *p* < 0.001). This was followed by the factors of patient cost reduction (mean difference = 0.33, *p* = 0.029), and informing patients and agreement between the two parties about any treatment change (mean difference = 0.32, *p* < 0.010).

### Communication

**Figure 5.**
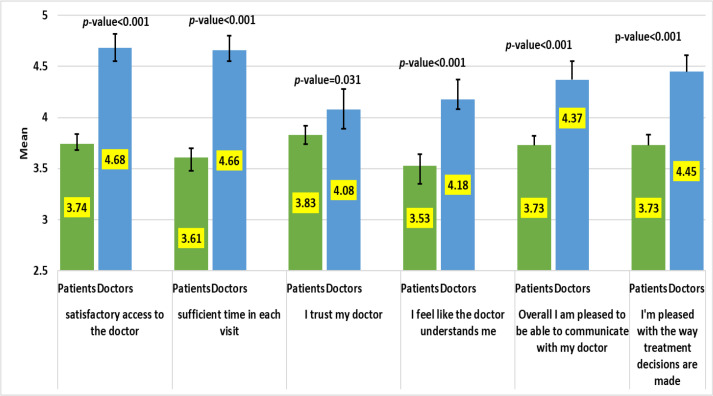
Factors influencing the quality of communication between patients and doctors. Note: p-values from t-tests of the mean difference between doctors and patients’ responses for each dimension.

*Figure 5* presents the results concerning the factors that influence the quality of communication between rheumatologists and patients in rheumatic diseases. Overall, there seems to be a high rate of disagreement between patients and rheumatologists regarding the quality of their communication. On average, patients assigned lower values in all dimensions compared with rheumatologists, and the differences in the mean ratings were all statistically significant. Specifically, statistically significant differences in the views between the groups were found regarding access (p<0.001), duration of visit (p<0.001), understanding (p<0.001), and overall communication (p<0.001).

**Figure 6.**
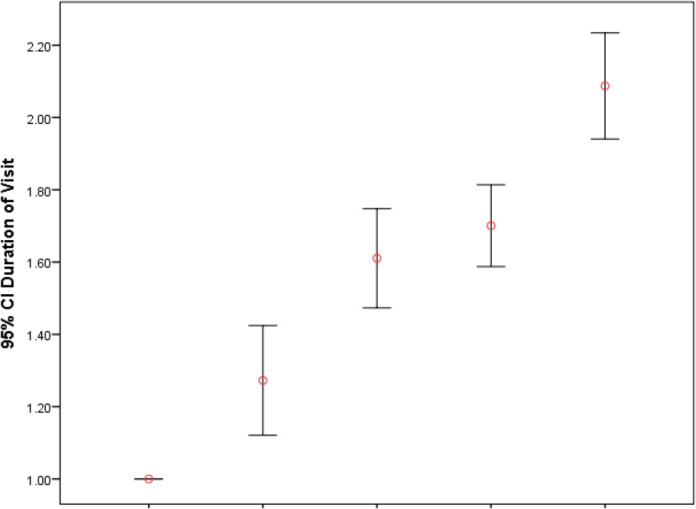
Relation between duration of visit and satisfactory communication with the Doctor.

An important factor influencing the doctor-patient relationship is the time devoted to patient. *Figure 6* presents the relationship between the average time per patient’s visit to the doctor, and the patient views on a successful communication with his/her doctor. It appears to be a positive exponential relationship between time and satisfactory communication with the doctor.

### Factor Analysis

Factor analysis was performed to investigate the interrelationship between long-term adherence and several construct for patients and doctors. The estimated Eigenvalues (EVs) (see *Supplemental Table 2* and *Supplemental Table 3*) represent the amount of variance in the constructs under investigation.^43^ Factors with EVs >1 were selected, according to the Kaiser-Guttman rule,^44,45^ in order to satisfy the following criteria: i) the matrix has to be positive, ii) definite, and iii) factorable.^46^ The cumulative percentage is 66.9% variance for the patients (*Table 2*) and 75.98% variance for the physicians (*Table 3*). For both parties, the most important factors contributing to long-term adherence are stable efficacy and safety of the therapeutic regimen.

**Table 2.** Initial Eigenvalues concerning factors contributing to long term adherence – Patients.

**Component**	**Initial Eigenvalues**		**Extraction Sums of Squared Loadings**			
	**Total**	**% of Variance**	**Cumulative %**	**Total**	**% of Variance**	**Cumulative %**
**Efficacy**	2,622	43,700	43,700	2,622	43,700	43,700
**Safety**	1,393	23,217	66,917	1,393	23,217	66,917
**Maintenance of a successful therapeutic regimen**	0,678	11,302	78,220			
**Adjustment of treatment**	0,551	9,189	87,408			
**Patient-side cost reduction**	0,470	7,839	95,248			
**Informing and agreeing between the two parties**	0,285	4,752	100,000			

Extraction Method: Principal Component Analysis.

**Table 3.** Initial Eigenvalues concerning factors contributing to long term adherence – Rheumatologists.

**Component**	**Initial Eigenvalues**			**Extraction Sums of Squared Loadings**		
	**Total**	**% of Variance**	**Cumulative %**	**Total**	**% of Variance**	**Cumulative %**
**Efficacy**	3,132	52,206	52,206	3,132	52,206	52,206
**Safety**	1,427	23,780	75,986	1,427	23,780	75,986
**Maintenance of a successful therapeutic regimen**	0,748	12,469	88,455			
**Adjustment of treatment**	0,346	5,768	94,223			
**Patient-side cost reduction**	0,194	3,234	97,458			
**Informing and agreeing between the two parties**	0,153	2,542	100,000			

Extraction Method: Principal Component Analysis.

**Figure 7a, 7b.**
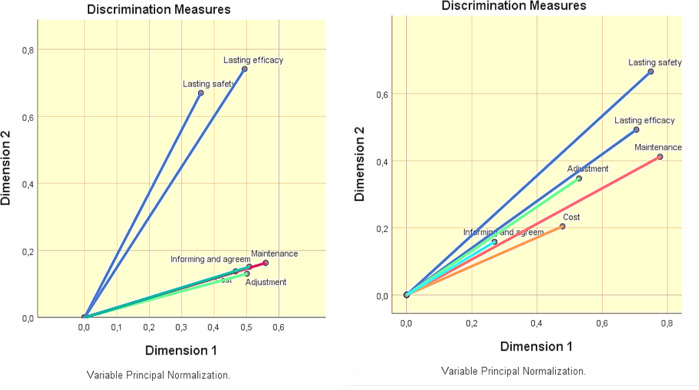
Diagrammatic presentation of factors contributing to Long Term Adherence for Patients (Figure 7a) and Rheumatologists (Figure 7b).

*Figure 7a* and *Figure 7b* provide a diagrammatic presentation of the most important factors contributing to Long-Term Adherence. Patients (*Figure 7a*) assign great values in safety and efficacy and much less to the rest of the factors. Rheumatologists (*Figure 7b*) have great interest for safety and efficacy but they also take into account other factors like the maintenance of a successful therapeutic regimen, adjustment of the treatment to their needs and the cost of therapy.

## DISCUSSION

Our current study indicates that medication-taking behaviour is extremely complex, requiring multifactorial strategies examining the different dimensions of doctor-patient relationship. The right choice of treatment contributes substantially to clinical outcomes and long-term adherence. Given the existence of alternative clinical scenarios, the right choice of treatment, would lead to a more or less adequate and successful adherence, and would eventually have an impact on the desired clinical outcome. Disease management and efficient utilization of resources contributes to improvements in patients’ quality of life and better health outcomes.

Rheumatologists in our study assigned high scores in attaining effectiveness, and, in particular, pain relief, joint swelling, mobility, and controlling stiffness. Ensuring safety and access were the major factors for both groups when it comes to the choice of treatment. It is obvious that an ineffective treatment needs to be changed. In this case, it imprints the fear of a less effective future treatment. In addition, we can see the need of motivation that both parties have, in order to remain adherent or to continue good communication. Interestingly, the route and frequency of treatment administration were the less important factors both for patients and rheumatologists regarding the choice of therapy.

Regarding long-term adherence, both groups indicated high level of convergence in their views in safety and efficacy. Factor analysis confirmed this finding. Rheumatologists emphasised the need to maintain the therapeutic regimen that contributes most to the achievement of desired clinical effects. Tapering of medication and cost were the less important factors both for patients and rheumatologists. Among all factors examined, the greatest discrepancy between patients and their treating physicians regarded to the quality of communication between them. Overall patients seem to be less satisfied in their communication with doctors. Patients reported the need for more time per visit, better access to medical services, and more qualitative channels of communication with their doctor.

The findings of our study are in agreement with previous studies,^18,20 ,21,23,31^ that patients’ concern about side effects of the therapeutic regimen, cost imposed to patient, changes of treatment, and barriers to communication contribute to sub-optimal levels in the doctor-patient relationship. In addition, they have an impact on health outcome, quality of life, and long-term adherence.^47,48^

Although various models have been suggested considering a closer collaboration between the two parts,^9–12^ barriers are raised because of the existing asymmetry in information. Lack of full understanding of the disease,^49^ lack of involvement in the treatment decision-making process,^16^ and suboptimal medical literacy^15^ are some factors that set patients in a disadvantageous position. Also, beliefs and attitudes concerning their previous medical experiences can affect the degree of medication adherence.^30,50,51^

Concerning the doctors’ side, they may unwittingly contribute to patient’s nonadherence due to lack of understanding between the two parts,^25^ by prescribing complex, difficult to read drug regimens,^17^ and inadequately considering the financial burden to the patients.^30,31^

Another factor affecting the whole procedure is the quality of communication between the two parts and the trust that patients show to their doctor. We must keep in mind that the doctor-patient relationship must count on both verbal and non-verbal communication, so the patient is capable of understanding illness, risks and benefits of the chosen treatment^30^ and feels encouraged to participate in the decision-making process^38,39,52,53^

Taking under consideration the above factors, the implementation of patient educational programs would minimize the existing discrepancies^54^ and improve substantially the doctor-patient relationship.^55^. Rheumatologists should also be trained by specialists (for example, psychologists or health economists) about the possible factors affecting short or long-term adherence, as well as by communication professionals in order to improve their communication skills. Additionally, safer and more reliable measures of adherence should be tested and implemented accordingly such as a pill-reminder system, regular blood tests or a Real Time System.

In a country like Greece that is characterized by limited resources and, in many cases, health need has turned to be catastrophic,^56^ a better doctor-patient relationship would lead to improvements in health outcome, better adherence, and more cost -effective therapies.
